# Patient pathways for rare liver diseases: a European template developed by ERN RARE-LIVER

**DOI:** 10.1186/s13023-026-04556-3

**Published:** 2026-09-05

**Authors:** Sarah Raevens, Renée Duijzer, Andreea Fodor, Matt Bolz Johnson, Nagham Issa, Sophie Pénisson, Samir Patel, Alice Bertolini, Carmen A. J. Teemer, Clemence Ramier, Laure Elkrief, Marco Senzolo, Virginia Hernandez-Gea, Ansgar W. Lohse, Bogdan Procopet, Joost P. H. Drenth, Aurélie Plessier

**Affiliations:** 1 Hepatology Research Unit, Department of Internal Medicine and Pediatrics, Liver Research Center, Ghent University, Ghent, Belgium; 2 Department of Gastroenterology and Hepatology, Ghent University Hospital, Ghent, Belgium; 3 Department of Gastroenterology and Hepatology, Radboudumc, Nijmegen, The Netherlands; 4 “Iuliu Hatieganu” University of Medicine and Pharmacy, Cluj-Napoca, Romania; 5 Department of Gastroenterology, “Prof Dr Octavian Fodor” Regional Institute of Gastroenterology and Hepatology, Cluj-Napoca, Romania; 6 EURORDIS - Rare Diseases Europe, Paris, France; 7 Department of Medicine, University Medical Centre Hamburg-Eppendorf, Hamburg, Germany; 8 AMVF French Patient’s Association for Vascular Liver Diseases, Service d’Hépatologie, DMU DIGEST, Centre de Référence des Maladies Vasculaires du Foie, FILFOIE, Clichy, France; 9 Gastroenterology and Multivisceral Transplant Unit, Padova University Hospital, Padova, Italy; 10 PKD Familiäre Zystennieren e.V., Hamburg, Germany; 11 Aix Marseille Univ, INSERM, IRD, SESSTIM, Sciences Économiques & Sociales de la Santé & Traitement de l’Information Médicale, ISSPAM, Marseille, France; 12 Service d’Hépato-Gastroentérologie, Hôpital Trousseau, CHRU de Tours, Tours, France; 13 Barcelona Hepatic Hemodynamics Laboratory, Liver Unit, Hospital Clinic, Institut d’Investigacions Biomèdiques August Pi i Sunyer (IDIBAPS), CIBEREHD (Centro de Investigacion Biomedica en Red Enfermedades Hepaticas y Digestivas) University of Barcelona, Barcelona, 08036 Spain; 14 Department of Gastroenterology and Hepatology, Amsterdam UMC, University of Amsterdam, Amsterdam, The Netherlands; 15 Service d’Hépatologie, DMU DIGEST, Centre de Référence des Maladies Vasculaires du Foie, FILFOIE, Centre de Recherche sur l’inflammation, Inserm, UMR 1149, Université de Paris, AP-HP, Hôpital Beaujon, 100 Bd du Général Leclerc, Clichy, Paris 92110 France; 16 European Reference Network of Rare Hepatological Diseases (ERN RARE-LIVER), Hamburg, Germany

**Keywords:** Patient pathway, Rare liver disease, Patient empowerment, Multidisciplinary care

## Abstract

**Background:**

Rare liver diseases are associated with diagnostic delay, fragmented care, inconsistent management, and limited patient support. Patient pathways may help address these challenges by translating clinical guidance and patient priorities into practical, coordinated care processes.

**Main body:**

This manuscript presents a European template for developing patient-centred pathways for rare liver diseases within ERN RARE-LIVER. The template was developed through multidisciplinary collaboration involving hepatologists, specialist nurses, patient representatives, patient organisations, and EURORDIS, and was informed by clinical guidance, patient-journey principles, and iterative expert and patient review. The proposed framework follows four phases of the patient journey: pre-diagnosis, diagnosis, management, and long-term follow-up. It includes disease-specific sections, educational resources, frequently asked questions, and “10 key questions” to support shared decision-making. Pathways for non-cirrhotic portal vein thrombosis, polycystic liver disease, and portosinusoidal vascular disorder illustrate the practical application of the template.

**Conclusion:**

This European template provides a practical, adaptable framework aimed at supporting harmonised, multidisciplinary, and patient-centred care for rare liver diseases across European healthcare settings. Future work should evaluate implementation, usability, and impact on care coordination and patient experience.

## Background

A disease is classified as ‘rare’ if it affects less than 1 in 2000 people [1]. Rare liver diseases pose unique challenges, such as diagnostic complexity, limited awareness among healthcare providers (HCPs), and fragmented care. These challenges often lead to delayed diagnosis, inconsistent treatment approaches, and gaps in patient education.

To address these barriers, patient pathways have become essential tools in healthcare delivery. A patient pathway is a structured, multidisciplinary plan that outlines the typical sequence and timing of medical care and interventions a patient should receive for a specific condition [2, 3]. Their primary purpose is to define how the health system should optimally address the needs of patients with specific conditions [4]. They achieve this by summarising existing guidelines, clinical protocols, and best practices to support healthcare professionals in the diagnosis, treatment, and follow-up care. Well-designed patient pathways promote coordinated care, ensuring care is consistent, efficient, and of high quality, while informing and empowering patients [5].

At the European Union (EU) level, member states are committed to ensuring equitable access to timely assessment, diagnosis and high-quality, cost-effective care for all individuals with rare diseases. This commitment also includes integrating rare diseases into broader social services and healthcare policies [6, 7]. In 2017, European Reference Networks (ERNs) [8], virtual networks of HCPs across the EU, were established to improve outcomes for patients with rare diseases [9]. The primary mission of ERNs is to improve care through a patient-centered approach that involves multiple stakeholders.

Individuals affected by rare liver diseases often face a substantial psychosocial burden, driven by diagnostic delays, chronic fatigue, depressive symptoms, and the emotional toll of navigating a complex medical landscape [10–13]. Accessing expert care, disease-related information, and social guidance remains difficult for a large proportion of patients [12, 14]. Patient pathways can help bridge this gap by structuring multidisciplinary care.

While overarching rare disease initiatives such as RarERN Path provide valuable general structures [15], rare liver diseases present distinct clinical complexities. This manuscript describes the rationale, principles, and practical framework endorsed by ERN RARE-LIVER for developing harmonised, hepatology-tailored patient pathways that operationalise and expand upon broader European models.

## Development process

Since 2019, EURORDIS – Rare Disease Europe [4] and the Joint Action on Integration of ERNs into National Healthcare Systems (JARDIN program) have been working collaboratively to implement the Patient Journey framework across various rare disease areas. This initiative aims to empower patients to contribute their lived experiences toward the development of care pathways, ensuring that services are more responsive to patient needs.

One of the aims of ERN RARE-LIVER has been to develop a patient pathway approach tailored to rare liver diseases. In this context, the network aligned its efforts with EURORDIS’ work on patient journeys to help ensure that clinical care reflects patient-identified priorities and challenges.

### Multidisciplinary core group and stakeholder engagement

To guide the development process, a dedicated core working group was established within ERN RARE-LIVER (SR, RD, AF, NI, SP, SP, AB, CT, AL, JD, AP). This group comprised hepatology nurse specialists, clinical experts, and patient representatives. Clinical experts and specialist nurses were selected through the established disease-specific working groups of ERN RARE-LIVER, ensuring balanced clinical expertise and broad geographic representation across European reference centers. Patient representatives and patient experts were invited through affiliated European patient advocacy organisations (PAOs) and national patient networks in collaboration with EURORDIS. The participating group included 4 patient representatives from 3 countries (France, Italy, Germany), ensuring broad geographic representation. A planning meeting was held with a representative from EURORDIS (MJB), who provided methodological guidance and helped structure the overall approach. Inclusion of diverse expertise was deliberate to ensure balanced integration of scientific evidence and patient perspectives.

Initial and ongoing working meetings facilitated an iterative, co-creative process. These sessions emphasised transparency, inclusivity, and collaboration, allowing continuous feedback from all stakeholders to refine pathway content and format.

### Literature review

A targeted narrative literature review was conducted to identify existing patient pathway frameworks, clinical guidelines, and best practices relevant to rare conditions. The review encompassed electronic databases including PubMed and Embase, covering publications from 2000 to 2024. Search terms combined concepts related to “patient pathways,” “rare diseases,” “liver diseases,” and “care coordination”. Inclusion criteria focused on peer-reviewed articles describing pathway development, implementation, or evaluation in rare or complex diseases. The purpose of this review was to inform pathway content and structure, not to provide a systematic evidence synthesis. The identified literature and published clinical guidelines helped define the overarching framework by highlighting common structural care-coordination gaps across rare conditions and establishing the essential clinical milestones required for standardized rare liver disease pathways.

### Patient and public involvement

Patient involvement was conducted as patient and public involvement within a service-development initiative, not as human-subject research. Patient experts and patient-organisation representatives contributed experiential knowledge, reviewed pathway drafts, and advised on patient priorities, communication needs, and usability. No intervention was performed, no clinical data were collected, and no identifiable personal data were analysed for research purposes. Formal research ethics approval was therefore not required according to applicable institutional rules. Contributors agreed to participate in the pathway-development process and to the use of aggregated, non-identifiable feedback to inform the pathways.

### Iterative review and endorsement

Draft pathways underwent iterative review. Scientific scrutiny was provided by the VAscular Liver DIsease Group (VALDIG) scientific committee and other experts in rare liver diseases (LE, MS, VHG, BP, JD) to ensure clinical accuracy, relevance, and evidence-based rigor. Concurrently, patient advocacy organisations (PAO) and representatives critically evaluated the pathways for clarity, usability, and alignment with patient needs. Feedback from these reviews was systematically incorporated, with documented revisions enhancing pathway quality and applicability.

## ERN RARE-LIVER pathway-development framework

### Plan of approach in designing patient pathways

**Fig. 1:**
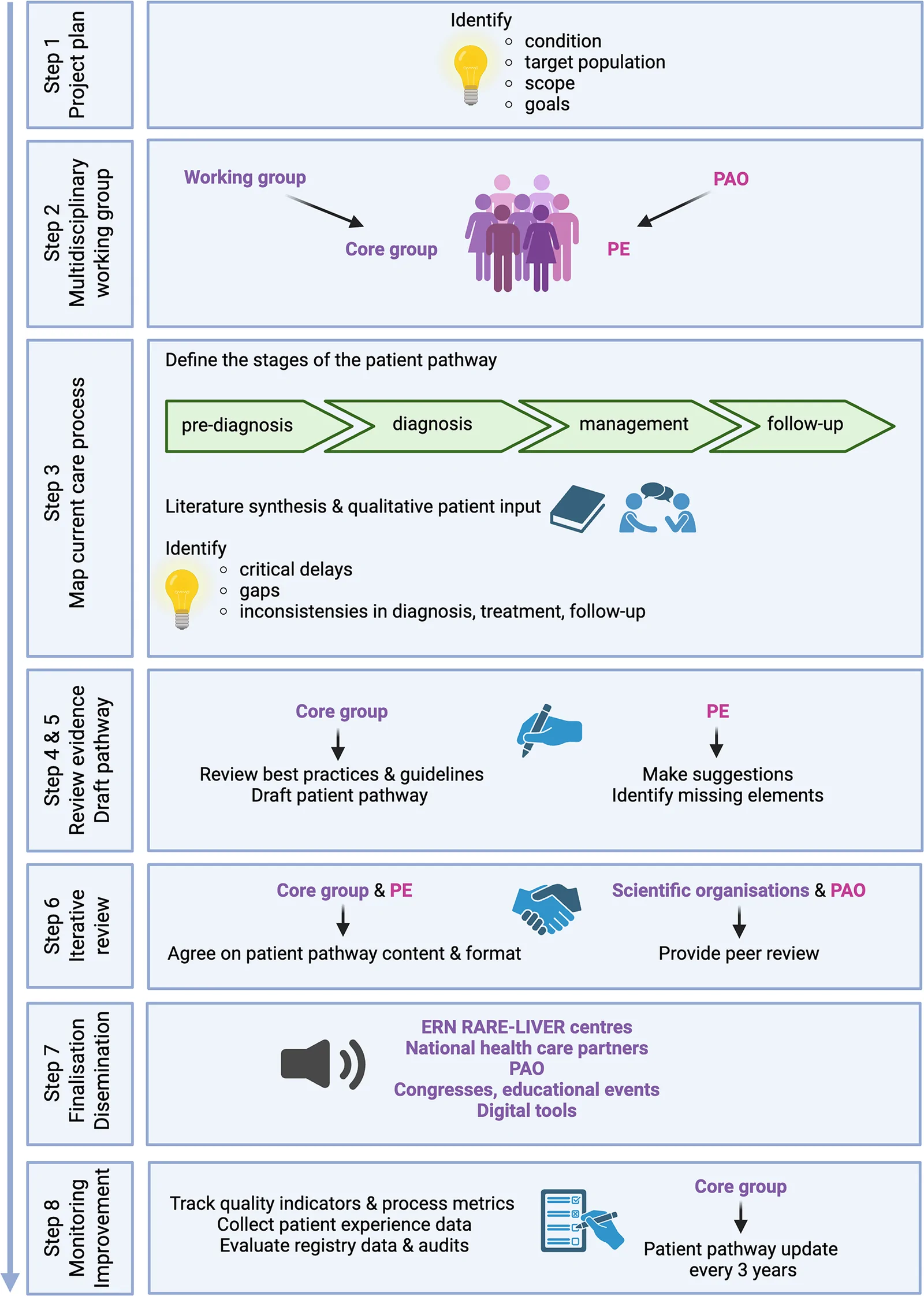
Summary of the stepwise approach to developing a patient pathway. Abbreviations: PAO, patient advocacy organization; PE, patient expert

The key steps in developing a patient pathway are summarised in Table 1 and visually outlined in Fig. 1.

**Table 1:** Key steps in developing a patient pathway

Key steps in developing a patient pathway
1. Project plan: define the scope and objectives
2. Establish a multidisciplinary working group
3. Mapping of the current care process
4. Review of the evidence and best practices
5. Draft of the ideal pathway
6. Iterative expert and patient review
7. Finalisation and dissemination
8. Monitoring and improvement

#### Step 1: Project plan

Developing a patient pathway starts with identifying the specific condition, target population, and goals such as improving diagnosis, standardising care, enhancing outcomes or patient education.

#### Step 2 & 3: Establish a multidisciplinary working group and mapping of the current care process

A multidisciplinary team is then assembled, including HCPs, patient representatives or associations, nurses and system stakeholders. The integration of their respective areas of expertise ensures that the pathway is informed by both evidence-based clinical standards and the nuanced insights drawn from patients’ lived experiences. More specifically, in this step, the core group contacts the appropriate PAO to include one or more patient experts (PE). During a first meeting, the goals of project are agreed on. Next, the content of the patient pathway was developed using relevant clinical guidance, expert discussion, and input from patient experts and patient organisations. This mapping effort identifies critical delays, gaps, and inconsistencies in diagnosis, treatment, and follow-up care.

#### Step 4 & 5: Review of the evidence and best practices, and draft of the ideal pathway

Based on this analysis, a comprehensive review of existing clinical guidelines and best practices is performed to inform the optimal care model. The patient pathway draft is subsequently developed, with attention to key stages such as symptom recognition, diagnostic algorithms, multidisciplinary treatment plans, and long-term follow-up protocols.

#### Step 6: Iterative expert and patient review

The group of PE reviews the patient pathway draft and generates suggestions for improving and solving missing elements. Some examples are: early referral to an expert centre, involvement of a psychologist, providing information on PAO, recommendations on travel, etc.

#### Step 7: Finalisation and dissemination

The core group finalises the patient pathway. If some suggestions are not retained, the reason for this should be communicated in a meeting, to agree on the final version by participating experts and patient representatives.

The final patient pathway is presented to involved scientific working groups and PAO for final review and endorsement.

Implementation is coordinated through ERN RARE-LIVER clinical centres, national healthcare partners, and PAO. Pathways are disseminated via professional networks, conferences, educational events, and online platforms. Integration with digital health tools, including telemedicine, remote monitoring, and patient-reported outcome platforms, represents a major future direction for extending access, particularly for patients in non-ERN centers.

#### Step 8: Monitoring and improvement

The care pathways are intended to be reviewed and updated every three years to incorporate emerging evidence and evolving clinical guidelines. To evaluate pathway adoption and its clinical impact across healthcare settings, a pragmatic framework of quality indicators is proposed. At the clinical level, centers are encouraged to monitor process metrics such as referral timelines, multidisciplinary team review rates, and adherence to disease-specific surveillance protocols. To assess patient experience and empower continuous improvement, brief annual satisfaction and usability surveys for patients and clinical staff will be implemented across ERN centers. Where feasible within existing registries, long-term evaluation may incorporate patient-reported outcome measures focusing on health-related quality of life (HRQoL) and fatigue. These combined feedback mechanisms will directly inform the triennial pathway revisions managed by the core working group.

### Examples of finalised patient pathways

Currently, detailed patient pathways for non-cirrhotic portal vein thrombosis, polycystic liver disease, and portosinusoidal vascular disorder have been developed. These resources are accessible through https://rare-liver.eu/training-education/patient-pathways. Patient pathway projects for other rare liver diseases, such as autoimmune hepatitis, primary biliary cholangitis, and Budd-Chiari syndrome, are currently under development.

### Creation of a generic patient pathway framework, core components and format

**Fig. 2:**
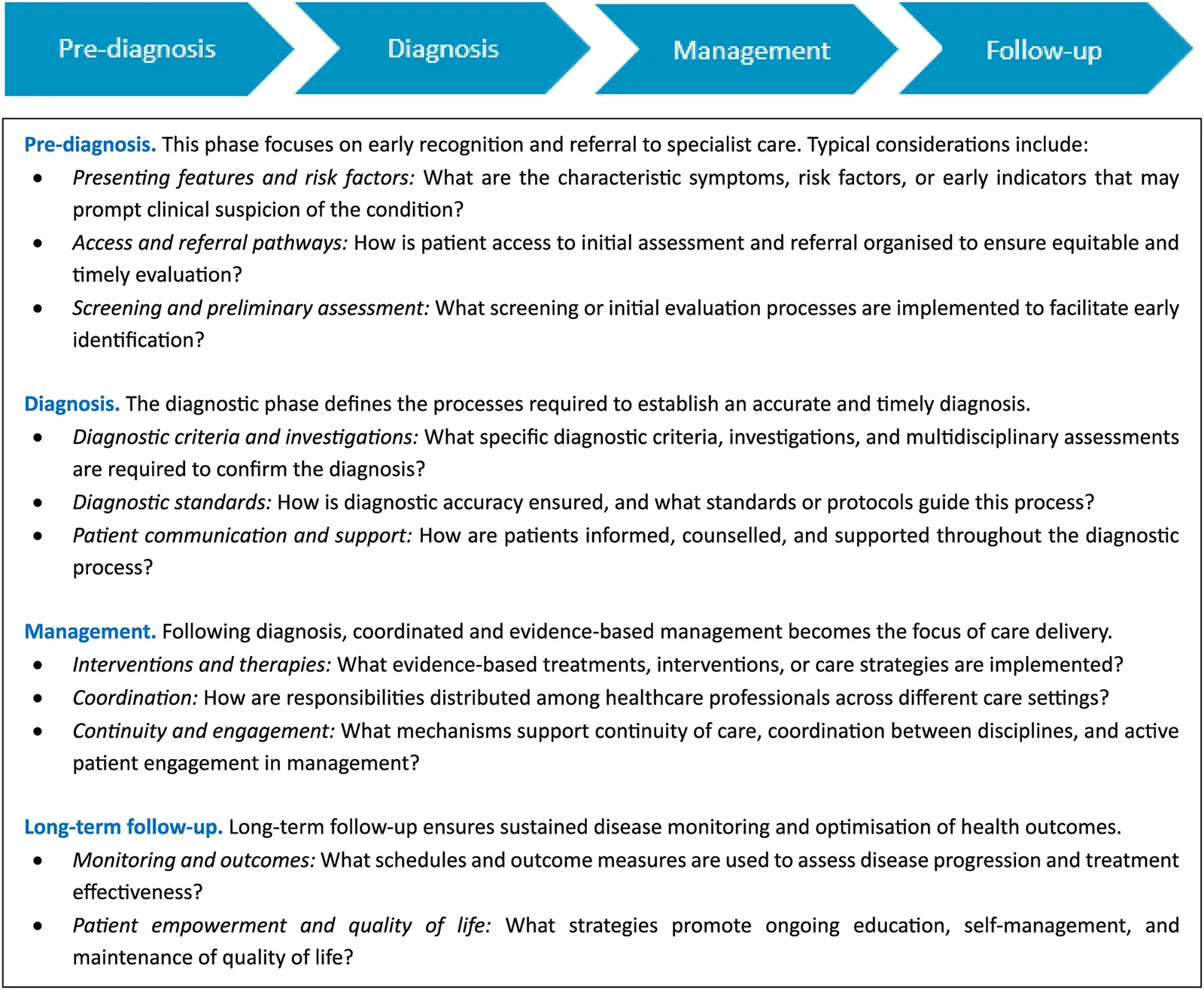
Generic patient pathway format for rare liver diseases

After designing individual disease pathways, the central objective of our initiative was to develop a generic patient pathway framework. This is an overarching structure (Fig. 2) which is designed to be adaptable across the spectrum of rare liver diseases. The introduction of such uniform structure promotes consistency, quality, and continuity of care.

**Fig. 3:**
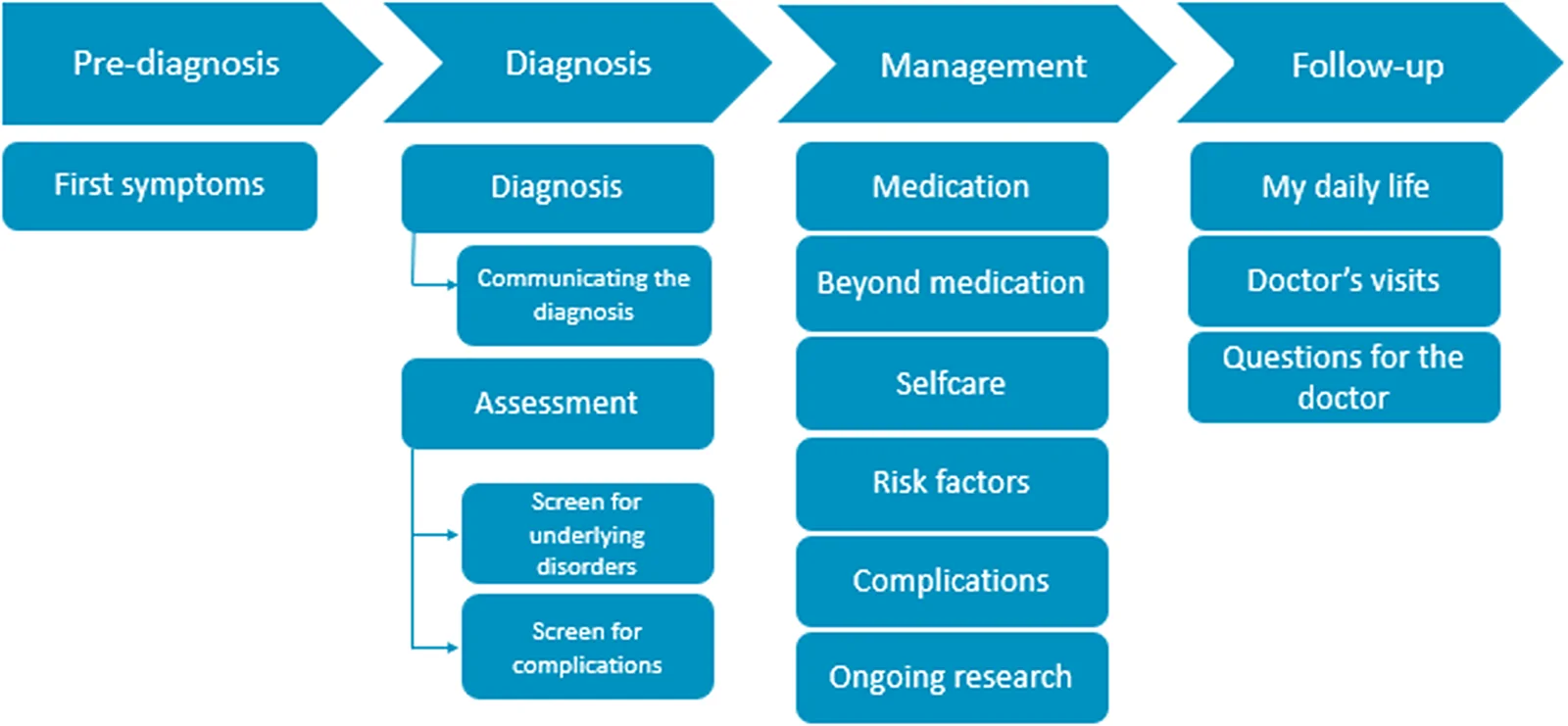
Example of disease-specific subsections in the patient pathway for non-cirrhotic portal vein thrombosis

Each care pathway is structured to follow the patient journey from pre-diagnosis through diagnosis, management, and long-term follow-up. Core components include symptom recognition and early detection protocols, diagnostic criteria and standardised investigations, coordinated multidisciplinary care plans, follow-up schedules, and strategies for transition of care. The framework is designed to be flexible and includes disease-specific subsections that allow for customisation, reflecting the unique clinical features and management needs of specific rare liver conditions (see Fig. 3).

**Fig. 4:**
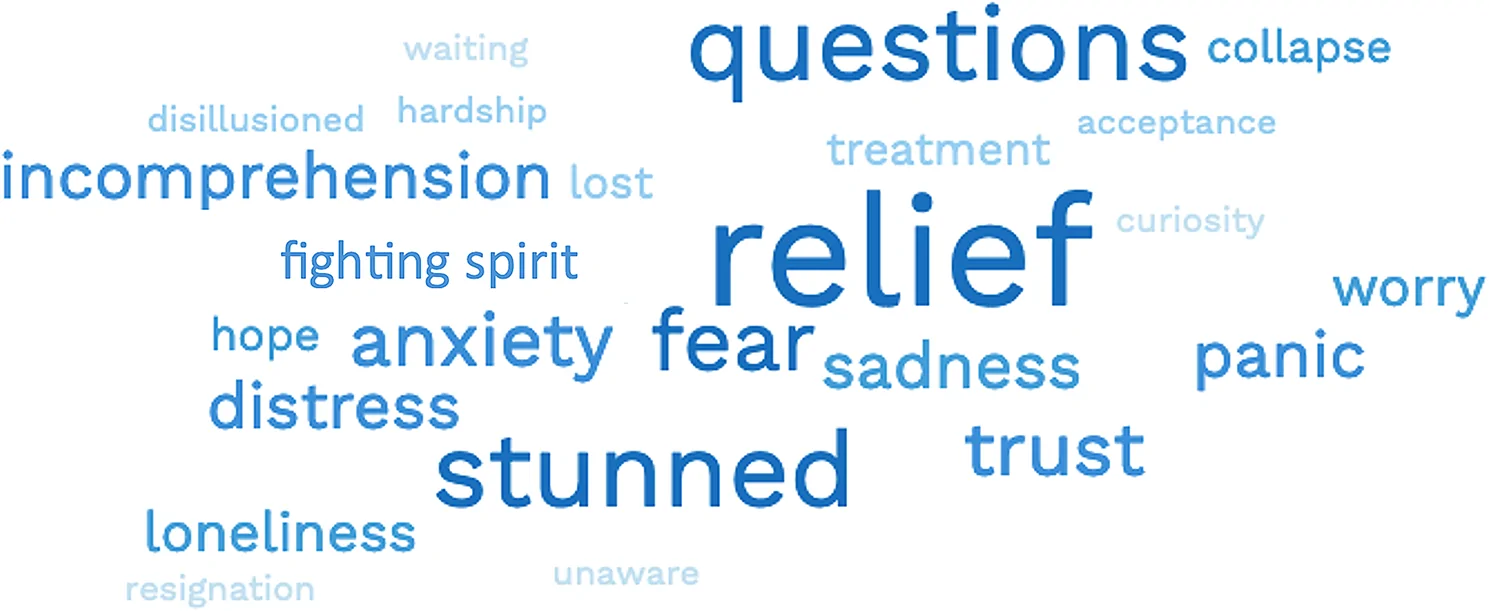
Example of anonymised, aggregated patient-organisation (AMVF) feedback on vascular liver disease diagnosis announcement, used to inform the pathway section on diagnostic disclosure. Word size reflects frequency of use

A critical aspect was the active engagement of patients, not only in reviewing content but in identifying the elements they consider indispensable to their care experience. Through targeted discussions and feedback sessions, patients emphasised the importance of addressing practical and psychosocial aspects of living with rare liver disease. These included HRQoL, lifestyle advice, and frequently asked questions about topics such as hereditary risk, pregnancy, work, and travel. As an example of patient-organisation input, AMVF shared anonymised, aggregated feedback from a community consultation on patients’ experiences of diagnostic disclosure (35 participants) (Fig. 4). This material was used to inform the communication and support elements of the pathway.

**Fig. 5:**
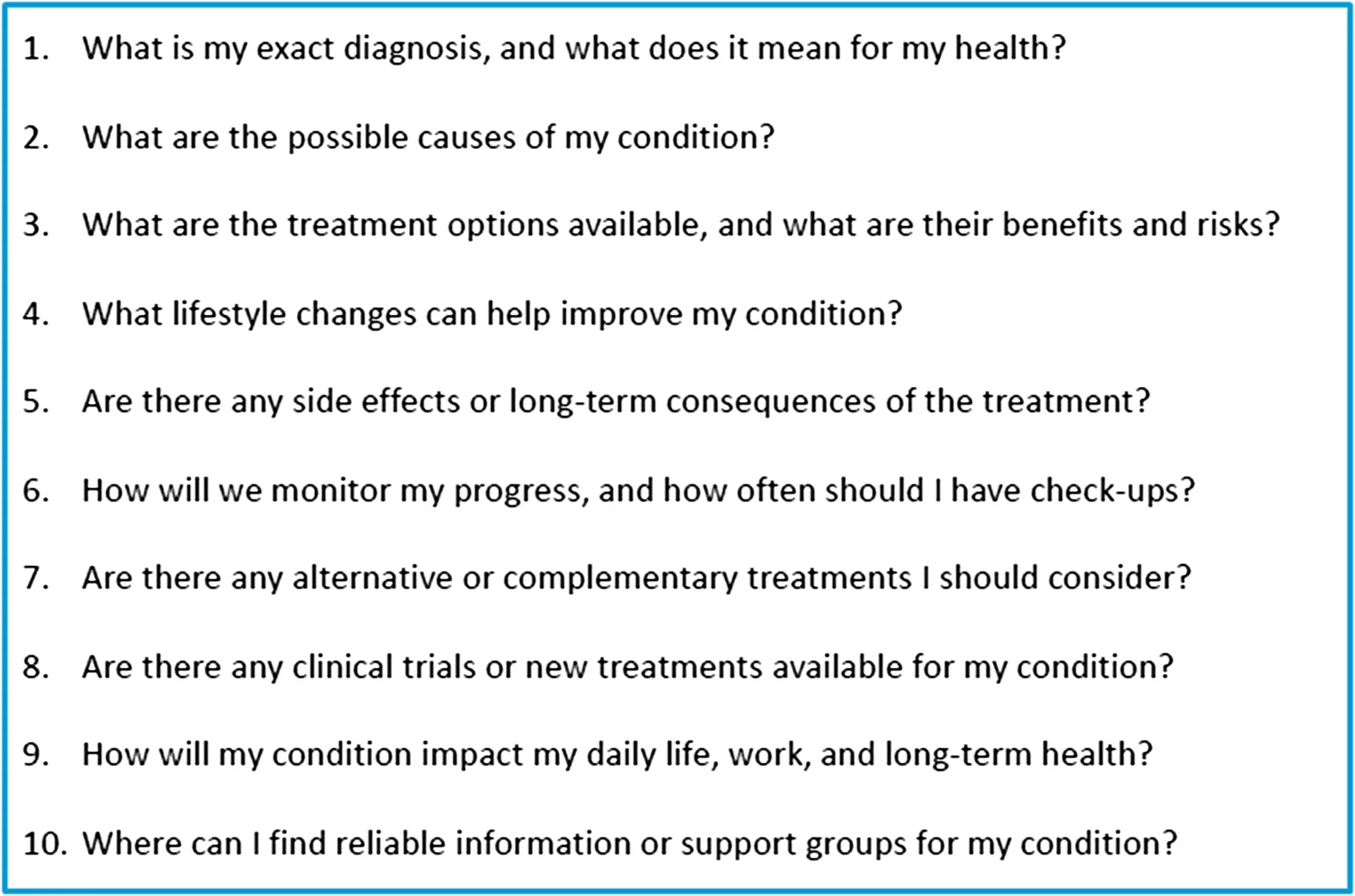
10 ‘’key questions” that patients are encouraged to ask their physician

To further support patient empowerment and shared decision-making, each pathway includes a set of “10 key questions” patients are encouraged to ask during medical consultations. This tool, adapted from a successful initiative developed by the Primary Biliary Cholangitis Working Group of ERN RARE-LIVER [16] (Fig. 5), is intended to promote meaningful dialogue between patients and healthcare professionals, and ensure patients receive clear, personalised information.

In addition, each pathway features a dedicated FAQ section covering everyday topics such as diet, physical activity, employment, family planning, and travel. Practical lifestyle guidance is provided to help patients make informed choices and maintain overall well-being in the context of their condition.

All patient pathways are made available online as interactive, patient-friendly PDFs, designed for ease of navigation and understanding. These digital resources incorporate clickable sections, visual aids, and regularly updated medical information. To maximise accessibility, the pathways will be translated into multiple languages, supporting patients, families, and healthcare professionals across different countries and regions.

## Discussion

This initiative represents the first pan-European, multidisciplinary patient pathway model for rare liver diseases, designed to bridge the gap between evidence-based guidelines and the real-world needs of patients [17, 18]. We developed a structured process to develop harmonised, patient-centred pathways for rare liver diseases. Each pathway follows the patient journey from pre-diagnosis to long-term follow-up, integrating evidence-based standards, multidisciplinary input, and patient perspectives. Core elements include early detection, standardised diagnostics, coordinated management, and follow-up strategies. Finalised pathways will be available online via ERN RARE-LIVER and updated regularly. Table 2 summarises the lessons learnt from our process and key attributes of successful patient pathway development.

By developing a standardised yet adaptable framework, multidisciplinary task forces synthesised existing literature and expert consensus, resulting in practical recommendations applicable across diverse healthcare settings. While recent national, European, and international guidelines have partially addressed gaps in care, significant unmet needs persist, including limited understanding of disease mechanisms and genotype–phenotype relationships, insufficient evidence for targeted therapies, and inadequate patient-reported outcome measures [19–26]. Developing patient pathways in parallel with clinical guidelines may further enhance implementation, ensuring that evidence-based recommendations are translated into practical, stepwise processes for care delivery and promoting consistency across multidisciplinary teams.

A central innovation of this pathway is the formal integration of patient perspectives alongside clinical guidance, addressing a critical gap in rare liver disease management. Health-related quality of life, psychosocial burden, and practical considerations, which are often overlooked in traditional guidelines, were systematically incorporated. Evidence from vascular liver diseases, primary biliary cholangitis, and polycystic liver disease highlights challenges such as fatigue, depression, and socioeconomic impact, particularly among women and vulnerable populations [12, 14, 27, 28]. Patient advocacy organisations contributed substantially to this initiative, shaping guidance on structured diagnostic disclosure and advocating for empathetic, patient-centred communication [11, 29, 30]. Incorporating patient-driven priorities and pragmatic advice on lifestyle, reproductive health, and hereditary risk enhanced both the relevance and usability of the pathways.

Compared to broader European rare disease tools (such as RarERN Path [15]), the primary novelty of our framework lies in its translation of complex hepatological guidelines into actionable, disease-specific workflows. By explicitly integrating liver-specific diagnostic milestones into a unified patient journey, this template addresses the unique clinical and operational realities of rare liver diseases.

Several lessons emerged from the development process of the patient pathways. Early and inclusive engagement of clinicians, allied health professionals, caregivers, and patients was essential to capture the full spectrum of care needs. Geographic diversity ensured sensitivity to differences in healthcare systems, specialist availability, and telemedicine capacity across Europe [28]. Flexibility in the framework allows adaptation to national and local contexts, accommodating emerging evidence and disease-specific nuances. Effective presentation, including visually oriented, user-friendly formats and interactive multilingual resources, improves comprehension, engagement, and equitable dissemination [31–34]. Although the collaborative design supported clinical accuracy, practical relevance, and alignment with patient priorities [35, 36], some challenges remain. Limited stakeholder diversity, overly complex pathways, and insufficient dissemination could hinder adoption, and pathways require regular updates to remain current. By anticipating these challenges, this initiative proposes a transferable framework for rare disease pathway development that balances clinical accuracy, patient relevance, and practical feasibility. It demonstrates the importance of sustained collaboration, continuous evaluation, and adaptability to achieve personalised, equitable, and sustainable care for individuals with rare liver diseases.

**Table 2:** Key attributes of successful patient pathway development

Key attributes of successful patient pathway development
1. Be patient-centered and incorporate patient-driven priorities
2. Be inter-/multidisciplinary
3. Ensure broad geographic representation to account for differences in healthcare organization
4. Represent best practice for most patients most of the time
5. Be applicable across the entire continuum of care
6. Pay attention to include advice on lifestyle, reproductive health, and hereditary risk
7. Provide visually oriented, user-friendly formats and interactive multilingual resources

### Limitations and future directions

Several considerations should be taken into account when interpreting and implementing this framework. The pathway template was developed within ERN RARE-LIVER and reflects the expertise of the clinicians, specialist nurses, patient representatives, patient organisations, and European partners involved. Although this provided multidisciplinary and geographically diverse input, local adaptation may be required to reflect differences in healthcare organisation, referral structures, resource availability, and patient-support networks across countries.

The evidence review was intended to inform the structure and content of the pathways rather than to serve as a formal systematic review, and the framework does not replace disease-specific clinical guidelines. In addition, while patient involvement was central to the development process, future work should continue to broaden patient and caregiver participation across countries and disease groups. Finally, implementation, usability, acceptability, and impact on care coordination and patient experience should be evaluated as the pathways are introduced and updated.

## Conclusions

In conclusion, this work contributes a comprehensive, harmonised patient pathway framework tailored to rare liver diseases. By systematically integrating patient experiences with clinical expertise, it promotes a structured foundation aimed at supporting consistent, patient-centered care across European reference centers. Continued implementation, evaluation, and iterative refinement will be essential to demonstrate its real-world impact on clinical care coordination and long-term patient outcomes.

## Data Availability

No datasets were generated or analysed during the current study.

## Abbreviations

AMVF: Association des maladies des vaisseaux du foie (patient association for vascular liver diseases)
ERN: European Reference Network
ERN RARE-LIVER: European Reference Network on Rare Hepatological Diseases
HCPs: Healthcare providers
HRQoL: Health-related quality of life
EU: European Union
JARDIN program: Joint Action on Integration of ERNs into National Healthcare Systems
PAO: Patient advocacy organization
PE: Patient expert
VALDIG: Vascular Liver Disease Group
